# Sediment Enzyme Activities and Microbial Community Diversity in an Oligotrophic Drinking Water Reservoir, Eastern China

**DOI:** 10.1371/journal.pone.0078571

**Published:** 2013-10-25

**Authors:** Haihan Zhang, Tinglin Huang, Tingting Liu

**Affiliations:** School of Environmental and Municipal Engineering, Xi’an University of Architecture & Technology, Xi’an, Shaanxi, China; Wageningen University, Netherlands

## Abstract

Drinking water reservoir plays a vital role in the security of urban water supply, yet little is known about microbial community diversity harbored in the sediment of this oligotrophic freshwater environmental ecosystem. In the present study, integrating community level physiological profiles (CLPPs), nested polymerase chain reaction (PCR)-denaturing gradient gel electrophoresis (DGGE) and clone sequence technologies, we examined the sediment urease and protease activities, bacterial community functional diversity, genetic diversity of bacterial and fungal communities in sediments from six sampling sites of Zhou cun drinking water reservoir, eastern China. The results showed that sediment urease activity was markedly distinct along the sites, ranged from 2.48 to 11.81 mg NH_3_-N/(g·24h). The highest average well color development (AWCD) was found in site C, indicating the highest metabolic activity of heterotrophic bacterial community. Principal component analysis (PCA) revealed tremendous differences in the functional (metabolic) diversity patterns of the sediment bacterial communities from different sites. Meanwhile, DGGE fingerprints also indicated spatial changes of genetic diversity of sediment bacterial and fungal communities. The sequence BLAST analysis of all the sediment samples found that *Comamonas* sp. was the dominant bacterial species harbored in site A. *Alternaria alternate*, *Allomyces macrogynus* and *Rhizophydium* sp. were most commonly detected fungal species in sediments of the Zhou cun drinking water reservoir. The results from this work provide new insights about the heterogeneity of sediment microbial community metabolic activity and genetic diversity in the oligotrophic drinking water reservoir.

## Introduction

In freshwater ecosystems, microbial communities harbored in the sediment play a pivotal role in biogeochemical cycling due to their involvement in transformation of nitrogen (N), phosphorus (P) and sulphur (S), organic matter demineralization and biochemical degradation [1-3]. Sediment microbes could enhance the adsorption of phosphorus [4]. Meanwhile, sediment microbial diversity and composition were strongly affected by hydrological regime fluctuation [5] and allochthonous organic carbon and nitrogen input process [6].

Numerous studies have been performed to determine the bacterial communities living in the sediments of various freshwater habitats, including eutrophic lake [6], athalassohaline lake [7,8], suboxic freshwater pond [9], ephemeral stream [10,11], spring [12] and intertidal wetland [13]. Those previous studies suggested that different freshwater environmental conditions harbored dramatically distinct microbial community composition. Drinking water reservoir is considerably different from these aquatic environmental ecosystems with distinguishing hydrological regime [14] and a significant part of the drinking water reservoir is characterized by poor nutrient flux to shape the particular microbial community structure. However, the metabolic and genetic characteristics of sediment microbial community in the oligotrophic drinking water reservoir are not comprehensively understood.

In the past few decades, a considerable amount of literature had primarily focused on pollution characteristics of heavy metals [15,16], polycyclic aromatic hydrocarbons [16], nitrogen and phosphorus adsorption and release [17], algae population and toxin [18] of the sediment in the drinking water reservoir. Recent study conducted by Röske et al. [19] evaluated sediment microbial diversity and composition in the mesotrophic drinking water reservoir Saidenbach using CARD-FISH and bar-coded pyrosequencing methods. However, most importantly, the sediment bacterial community activity and functional diversity of oligotrophic drinking water reservoir are still unclear.

Exploring the sediment microbial activity is important for understanding the sediment N and P quality and dynamics because of their momentous role in sediment nutrition biological transformation [20,21]. It is therefore methods for the quantitatively determination of sediment microbial activity, i.e., enzyme activity, have being widely employed to examine the ecological function of sediment microbial community [22]. Unfortunately, fewer studies have been determined sediment enzyme activity of the drinking water reservoir.

It is therefore the primary goal of the present work described here was to investigate sediment enzyme activity correlated to nitrogen metabolism and microbial community diversity in oligotrophic drinking water reservoir. The specific objectives of this study were to (1) determine the sediment urease and protease activities, and (2) evaluate the sediment bacterial community functional diversity and genetic diversity of bacterial and fungal communities from each six sampling sites located in the Zhou cun reservoir, eastern China.

## Materials and Methods

### Ethics statement

The sampling area is not privately-owned, so no specific permission was required for the described field studies. The field studies did not involve endangered or protected species. 

### Site description and sediment sampling

Zhou cun reservoir is located in Zao zhuang City, Shandong province, eastern China (34°56′N, 117°40′E). Zhou cun reservoir was built prior to 1985, which was oligotrophic with a maximum depth of 15-18 m, average depth of 13 m. It was one of the primary man-made public water supply reservoirs for Zao zhuang City. In June 2012, a global position system was used to locate the specific sampling positions (Table 1). 

**Table 1:** Location (longitude and latitude) and water depth of each sampling sites (site A, site B, site C, site D, site E, site F) in the Zhou cun drinking water reservoir, eastern China.

Sampling sites	Longitude (E)	Latitude (N)	Water depth (m)
Site A	117°41′12′′	34°56′31′′	15.0
Site B	117°40′55′′	34°56′39′′	12.0
Site C	117°40′40′′	34°56′51′′	8.0
Site D	117°40′17′′	34°56′28′′	10.0
Site E	117°40′18′′	34°56′56′′	8.5
Site F	117°40′04′′	34°57′01′′	7.5

Three replicate samples were respectively collected from each of six sampling sites using a Petersen stainless steel grab sampler [23]. At each site, sediment was collected from a top 30 cm layer of sediment [23]. After sampling, the sediment samples were placed into sterilized bottles, sealed, kept in the cooler filled with ice and immediately transported to the laboratory within 12 h. In the laboratory, each sediment sample was then divided into two parts, one part was stored at 4°C for enzyme activity and BIOLOG assay no more than 48 h, the other part was stored at -20 °C for sediment total DNA extraction.

### Sediment enzyme activities determination

To understand the sediment enzyme activity in Zhou cun reservoir, two sediment enzymes that are components of nitrogen cycles were chosen for this research. According to the method described by Guan Songyin [24] and little modification, sediment urease and protease activities were determined. Sediment urease activity was examined spectrophotometrically using urea as the substrate at 578 nm (UVmini-1240, Shimadzu, Japan), and the result was expressed as mg NH_3_-N/(g·24h). Protease activity was measured at 490 nm using the spectrophotometer (UVmini-1240, Shimadzu, Japan). Protease activity was expressed as mg NH_2_-N/(g·24h). The same procedure was followed for the control assays, with the exception that the substrate and sediment were added. Sediment enzyme activities were examined with replicates (*n*=3). 

### Sediment bacterial community functional diversity determination

To determine the fingerprint of sediment bacterial community functional diversity, BIOLOG-ECO plate (BIOLOG, Inc., Hayward, California, USA) with 31 sole carbon sources was employed [25]. Briefly, 5 grams sediment (d.w.) from each sampling sites was added to 45 ml sterilized NaCl solution (0.85%, w/v) and shook at room temperature with 120 rpm for 30 min. After standing for 30 min, the sediment suspension was diluted to 10^-3^, and 150 μl was added into each plate well using the eight channel pipettes (Bio-Rad, USA). The inoculated ECO plates were packed into polyethylene bags to reduce desiccation while incubating at 25°C in darkness. The absorbance (OD_590nm_) of wells was recorded for 240 h using ELISA plate reader every 24 h interval, and the results obtained at 144 h were used for diversity index and principal component analysis (PCA). Bacterial community activity in each ECO plate was expressed as average well color development (AWCD) [26]. Functional diversity index including Species richness (*R*) and Shannon’s Diversity (*H*) were measured [25,26]. The BIOLOG assay was reproduced in triplicate.

### Sediment DNA extraction and nested PCR amplification

To determine the sediment DNA and PCR amplification, sediment total microbial DNA was extracted using the Soil DNA Kit (Omega, USA) according to the manufacturer’s recommendations. The quality of extracted DNA was detected by electrophoresis in 1% agarose gels running in 1.0×Tris-Acetate-EDTA buffer. The nested PCR was used for 16S ribosomal RNA (rRNA) -V3 and 18S rRNA-ITS region amplification using a Thermal Cycler C1000 (Bio-Rad, USA). Sediment bacterial 16S V3 and fungal ITS regions were PCR amplified using the specific primers listed in Table 2. 

**Table 2:** Primer sets and nested PCR amplification regimes used in the nested PCR-DGGE analyses for sediment bacterial and fungal species in the Zhou cun drinking water reservoir, eastern China.

Nested PCR	Target	Primer set name and sequence 5′-3′	PCR amplification
First round	Bacteria [33]	fD1: AGAGTTTGATCCTGGCTCAG rP1: ACGGTTACCTTGTTACGACTT	94°C for 3 min, 30 cycles (94°C for 1min, 54°C for 1min, 72°C for 90s), 72 °C for 7min, 12 °C for ever
Second round	Bacteria [33]	341F-GC: ^*^ CCTACGGGAGGCAGCAG 534R: ATTACCGCGGCTGCTGG	94°C for 3 min, 30 cycles (94°C for 30s, 55°C for 30s, 72°C for 30s), 72 °C for 5min, 12 °C for ever
First round	Fungi [33]	ITS1F:CTTGGTCATTTAGAGGAAGTAA ITS4:TCCTCCGCTTATTGATATGC	94°C for 5 min, 35 cycles (94°C for 30s, 55°C for 30s, 72°C for 30s), 72 °C for 5min, 12 °C for ever
Second round	Fungi [33]	ITS1F-GC:^*^ CTTGGTCATTTAGAGGAAGTAA ITS2:GCTGCGTTCTTCATCGATGC	94°C for 5 min, 35 cycles (94°C for 30s, 55°C for 30s, 72°C for 30s), 72 °C for 5min, 12 °C for ever

*The GC clamp CGCCCGCCGCGCGCGGCGGGCGGGGCGGGGGCACGGGGGG was added to the 5′ end of the primer 341F and ITS1F

PCR reactions were performed in 50 μl volumes containing 25μl PCR MIX (Beijing Kangwei Century Company, China), 1μl primers, 22 μl H_2_O and 1μl DNA template. The second round PCR products were electrophoresed in a 1.0 % agarose gel, stained with Gel Rad™, and quantified using a standard DNA maker DL 2000 (Beijing Kangwei Century Company, China). The PCR products were stored at -20°C for the following DGGE analysis.

### Denaturing Gradient Gel Electrophoresis (DGGE) and clone sequencing

To explore the genetic diversity of sediment bacterial and fungal communities, D-code Universal Mutation Detection System (Bio-Rad, USA) was used for DGGE fingerprints analyses, which using the denaturing gradient ranging from 30% to 70% for bacterial and fungal PCR samples. 100% denaturant was defined as 7 M urea (Sigma) and 40% deionized formamide (Sigma, F-9037). For each sample, 45μl PCR production was loaded in the gel. Electrophoreses were run on 70 V for 13 h at 58°C in 1.0×Tris-Acetate-EDTA (TAE) buffer. The DGGE gel was then stained in Gel Red™ for 15 min, cleaned in sterilized water and scanned (Gel Doc XR, Bio-Rad, USA). Banding patterns of DGGE fingerprints were analyzed by Quantity One software Version 4.5 (Bio-Rad, USA). The peak density (relative intensity) of each band in the lanes of the gel was recorded for redundancy analyses (RDA). To understand more specifically of the sediment bacterial and fungal species living in Zhou cun drinking water reservoir, the examination of dominant DGGE band sequences was conducted. Specificity bands were excluded from the gel, then put them into the sterilized PCR tube with 30 μl water (MillQ, USA) and stored at 4°C overnight. 341f and 534r, ITS1F and ITS2 were used for bacterial and fungal amplification, PCR fragments were ligated into pMD 19-T vectors (TaKaRa, Japan) by the manufacturer’s instructions. Extraction plasmid from DH5α cells and sequenced with an ABI automated sequencer (Sangon Biotech, Shanghai, Co., Ltd, China). The clone sequences of bacterial and fungal species determined in this study from the DGGE gels of the sediments were submitted to the National Center for Biotechnology Information (NCBI) database under accession numbers as following: KC775757, KC775759 - KC775765 for bacterial community and KC775758, KC775766 - KC775770 for fungal community. Phylogenetic trees were constructed with the neighbor-joining (NJ) method with 1000 replicates to produce bootstrap values using the MEGA (version 5.05) software packages for windows. The sequences were classified with the least common ancestor (LCA) method based on the different taxonomies hosted by SILVA database.

### Statistical analysis

One-way ANOVA with Tukey-Kramer HSD test was performed to evaluate the significant differences of enzyme activities, *AWCD*, Species richness (*R*) and Shannon’s diversity (*H*) among sediment samples from different sites using SAS version 8.1 software (SAS Institute Inc., Cary, NC, USA). Principal component analyses (PCA) and redundancy analyses (RDA) were employed to analyze the BIOLOG and DGGE fingerprint data. RDA ordination axes (RDA1 and RDA2) were constrained to be linear combinations of environmental variables, thus allowing direct assessment of the relationship between environmental variables (urease and protease activities) and variation in the DGGE band relative intensities data using the software CANOCO for Windows (version 4.5) and CANODRAW.

## Results and Discussion

### Sediment enzyme activity

Sediment enzymes play a critical role in shaping the health degree and nutritional status of an aquatic water body. In this study, significant differences of enzyme activities were observed in urease activity ranging from 2.48 mg NH_3_-N/(g·24h) to 11.81 mg NH_3_-N/(g·24h). The highest urease activity was observed in site D, which was significantly higher than in site B (*F*=27.11, *P*<0.01). Proteases can hydrolyze proteins to amino acids. Protease activity in site D was 54.74 mg NH_2_-N/(g·24h), which was significant higher than that of site A and site F (*F*=358.41, *P*<0.01) (Table 3). 

**Table 3:** Urease and protease activities in the sediments from each six sampling sites (site A, site B, site C, site D, site E, site F) in the Zhou cun drinking water reservoir, eastern China.

Sampling sites	Urease activity [mg NH_3_-N/(g·24h)]	Protease activity [mg NH_2_-N/(g·24h)]
Site A	2.48±0.57C	17.41±0.16E
Site B	4.23±0.84BC	24.83±0.37D
Site C	7.61±0.73B	31.57±1.21C
Site D	11.81±0.67A	54.74±0.28A
Site E	11.50±1.10A	48.46±1.32B
Site F	5.80±0.00BC	16.28±1.01E
One way ANOVA	*F*=27.11, *P*<0.01	*F*=358.41, *P*<0.01

The data shown are the means and standard error (*n*=3). Values in the column followed by a different capital letter are significantly different by Tukey-Kramer HSD (*P*<0.01)

In the past decades, there have been massive researches on the sediment of aquatic environment; however, the sediment enzyme and microbial community functional and genetic diversity remain poorly understood. Sediment enzyme activities have been employed as potential indicators of sediment biological quality and nutrient dynamics, for example, urease activity was positively correlated to calcium carbonate deposit, and the removal rate of total nitrogen (TN).

### Sediment bacterial community functional diversity

**Figure 1:**
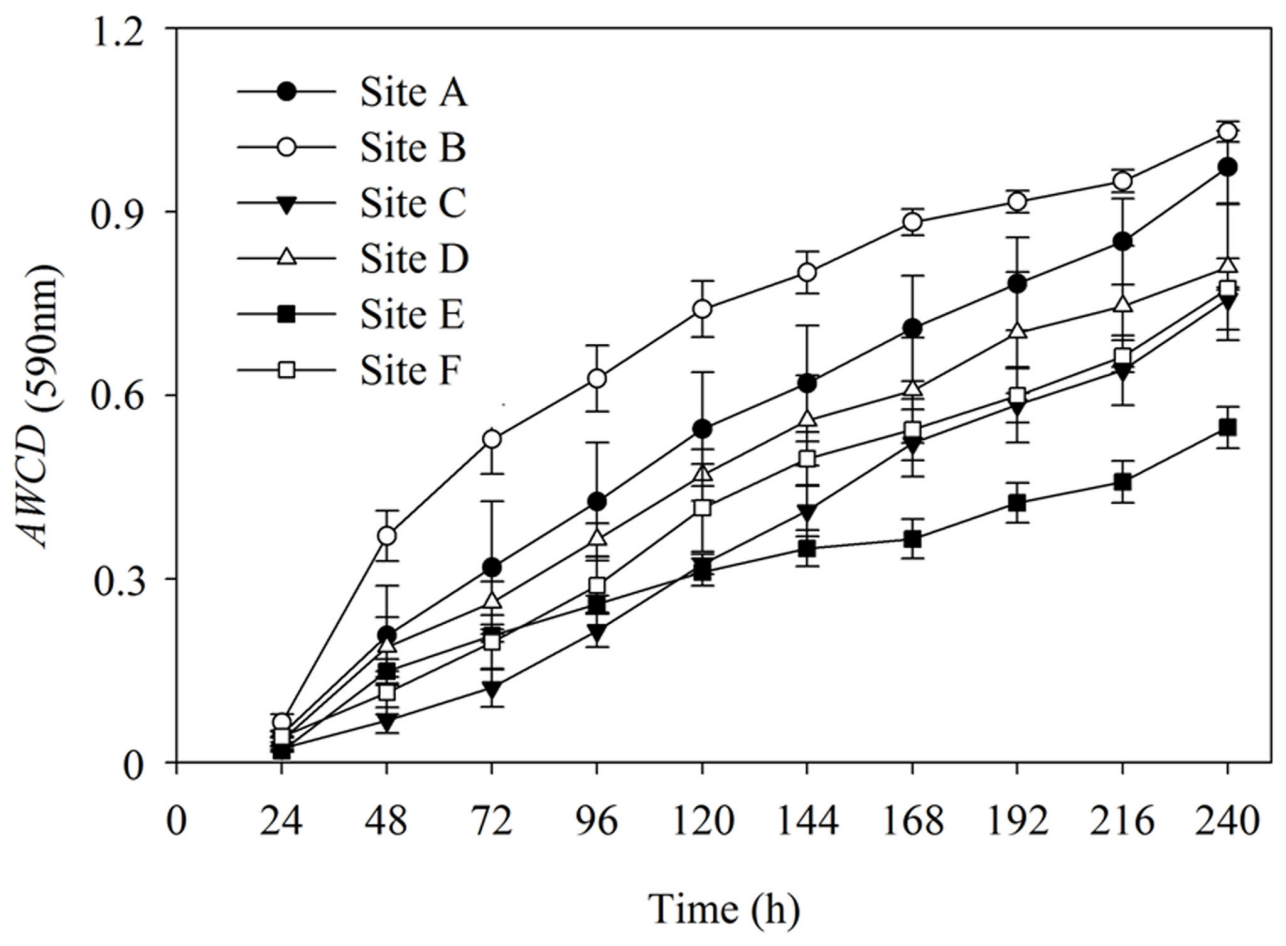
Kinetics of average well color development (*AWCD*_590nm_) curve of sediment bacterial communities. Sediments were collected from each six sampling sites (site A, site B, site C, site D, site E, site F). The data shown are the means and standard error (S.E) (*n*=3).

Microbial properties reflecting the microbial activity and diversity have great potential as bioindicators of the sediment because sediment microorganisms drive critical geochemical circulation in drinking water reservoir aquatic ecosystems. In this research, BIOLOG approach is used to improve our understanding of the biogeochemical functioning of sediment microbial community in the Zhou cun drinking water reservoir, eastern China. As shown in Figure 1, average well color development (*AWCD*_590nm_) of sediment bacterial community was increased steadily. 

**Figure 2:**
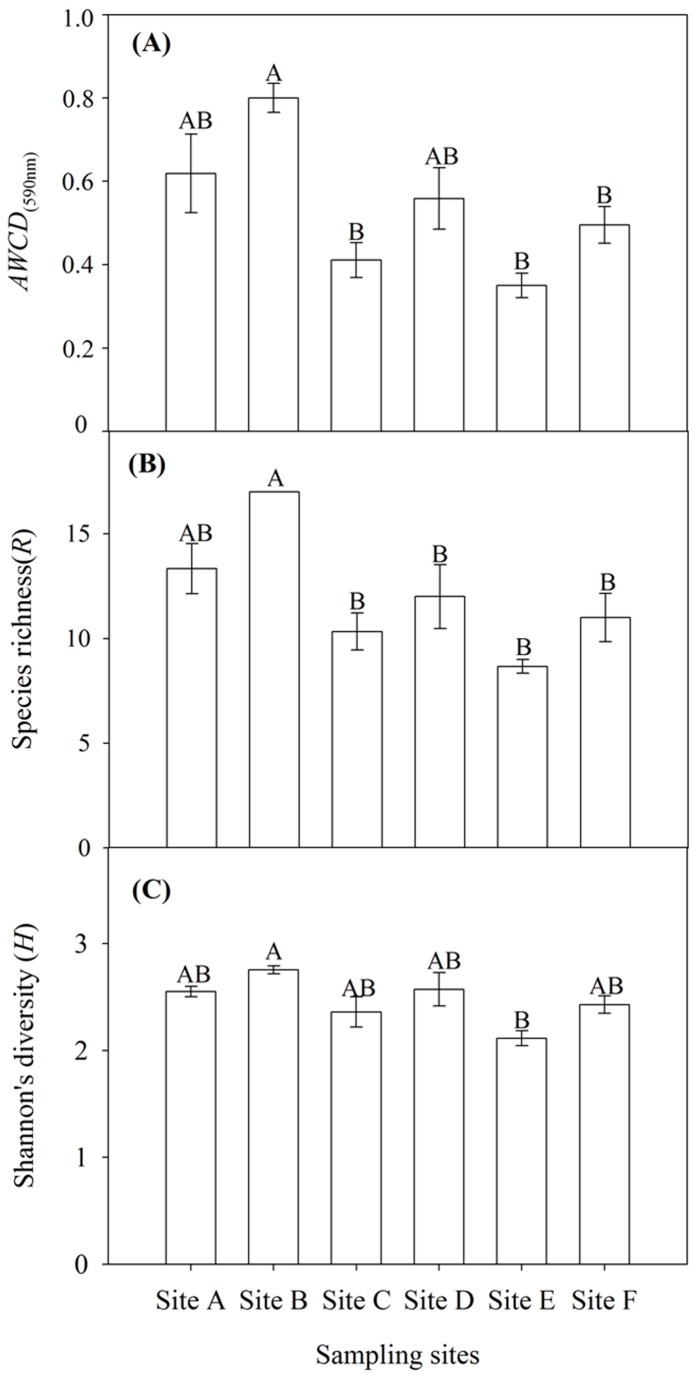
Functional diversity index of sediment bacterial community. (A) *AWCD*_(590nm)_, (B) Species richness (*R*) and (C) Shannon’s diversity (*H*) of bacterial community in the sediments from each six sampling sites (site A, site B, site C, site D, site E, site F) in the Zhou cun drinking water reservoir, eastern China. Bars followed by the same capital letter indicate no significant difference by Tukey-Kramer HSD (*P*<0.05). The data shown are the means and standard error (S.E) (*n*=3).

*AWCD* was an indicator reflecting carbon source utilization ability by bacterial community. The highest *AWCD* was observed in site B, which is significant higher than that of site D, E and F (*F*=7.69, *P*<0.01) (Figure 2 A). Species richness (*R*) and Shannon’s diversity (*H*) calculated using the data from the 144 h incubation readings were shown in Figure 2. Species richness (*R*) was ranging from 0.80 to 0.35. There was significant different between site B and site E (*F*=8.33, *P*<0.01) (Figure 2B). Shannon’s diversity (*H*) in site B was 2.76, which was significant higher than that of site E (*F*=4.77, *P*<0.05) (Figure 2C). Utilizations of the carboxylic acids did not significantly change with sampling sites (Table 4). Site B was different from other sites. The water quality such as dissolved oxygen, total nitrogen may influence the bacterial community activity. 

**Table 4:** Variance analysis of utilization of the six groups of carbon sources (carbohydrates, carboxylic acids, amino acids, polymers, amines and phenolic compounds) located in BIOLOG ECO plate by sediment bacterial community from each six sampling sites (site A, site B, site C, site D, site E, site F ) in the Zhou cun drinking water reservoir, eastern China.

Sampling sites	Carbohydrates	Carboxylic acids	Amino acids	Polymers	Amines	Phenolic compounds
Site A	0.354±0.208AB	0.460±0.041AB	0.579±0.102AB	0.650±0.282AB	0.055±0.026B	0.137±0.105AB
Site B	0.650±0.037A	0.371±0.084A	0.797±0.220A	1.241±0.135A	0.259±0.043AB	0.033±0.023AB
Site C	0.146±0.082B	0.306±0.054AB	0.246±0.034AB	0.367±0.084B	0.040±0.033B	0.026±0.015AB
Site D	0.208±0.044AB	0.414±0.078AB	0.526±0.062AB	0.458±0.055B	0.633±0.212A	0.022±0.004AB
Site E	0.498±0.038AB	0.258±0.060B	0.110±0.056B	0.040±0.009B	0.197±0.184AB	0.012±0.010B
Site F	0.223±0.044AB	0.208±0.051AB	0.391±0.218AB	0.500±0.194B	0.024±0.005B	0.440±0.190A
One way ANOVA	*F*=4.01*	*F*=2.31NS	*F*=3.17*	*F*=6.54**	*F*=3.92*	*F*=3.50*

The data shown are the means and standard error (*n*=3). Values in the same column followed by the different capital letter are significantly different by Tukey-Kramer HSD (*P* < 0.05)

*By analysis of variance: *P* < 0.05

** By analysis of variance: *P* < 0.01

NS, not significant

**Figure 3:**
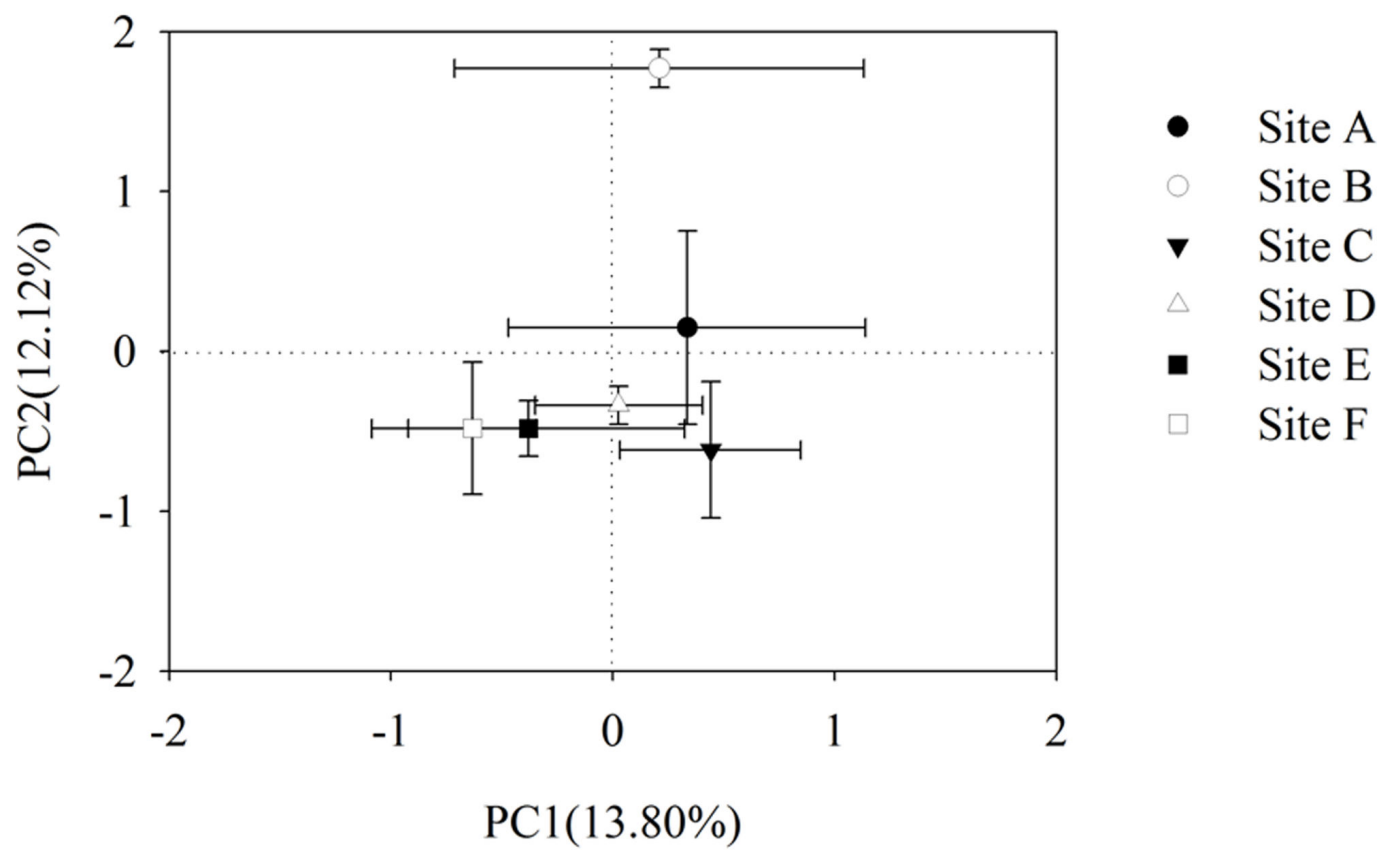
Principle component analyses (PCA) of bacterial functional diversity. Sediments were collected from each six sampling sites (site A, site B, site C, site D, site E, site F) in the Zhou cun drinking water reservoir, eastern China. Data were calculated based on sole carbon substrate utilization pattern using BIOLOG ECO micro plates after incubation of 144 h. Numbers in brackets represent the percentage of variation explained by each factor, PC1 explains 13.80% of the variance of the data and PC2 explains 12.12% of the variance in the data, respectively. Bars represent plus one standard error (S.E) (*n*=3).

The first two PCs (PC1 and PC2) explained 25.92% of the total variance in *AWCD* data (Figure 3). The PCA further revealed that metabolic profiles from the five sites (site A, C, D, E and F) were similar to each other, but significantly distinguished from that of site B (*P*<0.05). Separation of the five sites along PC2 was significantly affected by carbohydrates and carboxylic acids (data not shown). 

BIOLOG is a useful technique to explore aquatic microbial community metabolic characteristics [27]. Choi and Dobbs [27] compared the BIOLOG ECO and GN microplates abilities to distinguish among six aquatic environmental conditions, and PCA was used to analyses the BIOLOG data, and suggested that GN and ECO plates had the same ability to discriminate the bacterial community harbored in different aquatic environmental ecosystems. In this work, BIOLOG ECO plates were explored to examine sediment bacterial community. In this oligotrophic aquatic environmental condition, sediment bacterial community activities are higher than that of oligotrophic soil conditions [25]. Previous studies provide information about the sediment bacterial community functional diversity. For example, Andrea et al. [28] used BIOLOG GN_2_ plates containing 95 different sole carbon sources to examine the bacterial community activities in the sediment of Lake Velencei, Hungary, the results indicated that sediment microbial communities showed seasonal variations of the utilized carbon sources. Similarly, Biggs et al. [29] also used BIOLOG-ECO to study the influence of temperatures on the functional diversity of bacterial communities in sewer sediment, and revealed that different temperatures have different bacterial community metabolic profiles. Tiquia [30] examined the metabolic functional diversity of the heterotrophic bacterial community of the Rouge River from different sampling sites, and found that bacterial community functional diversity was related to physico-chemical characteristics such as dissolved oxygen and chlorophyll concentrations suggested that BIOLOG is powerful in monitoring metabolic functional diversity between different microbial communities. 

### DGGE profiles of sediment bacterial and fungal community

**Figure 4:**
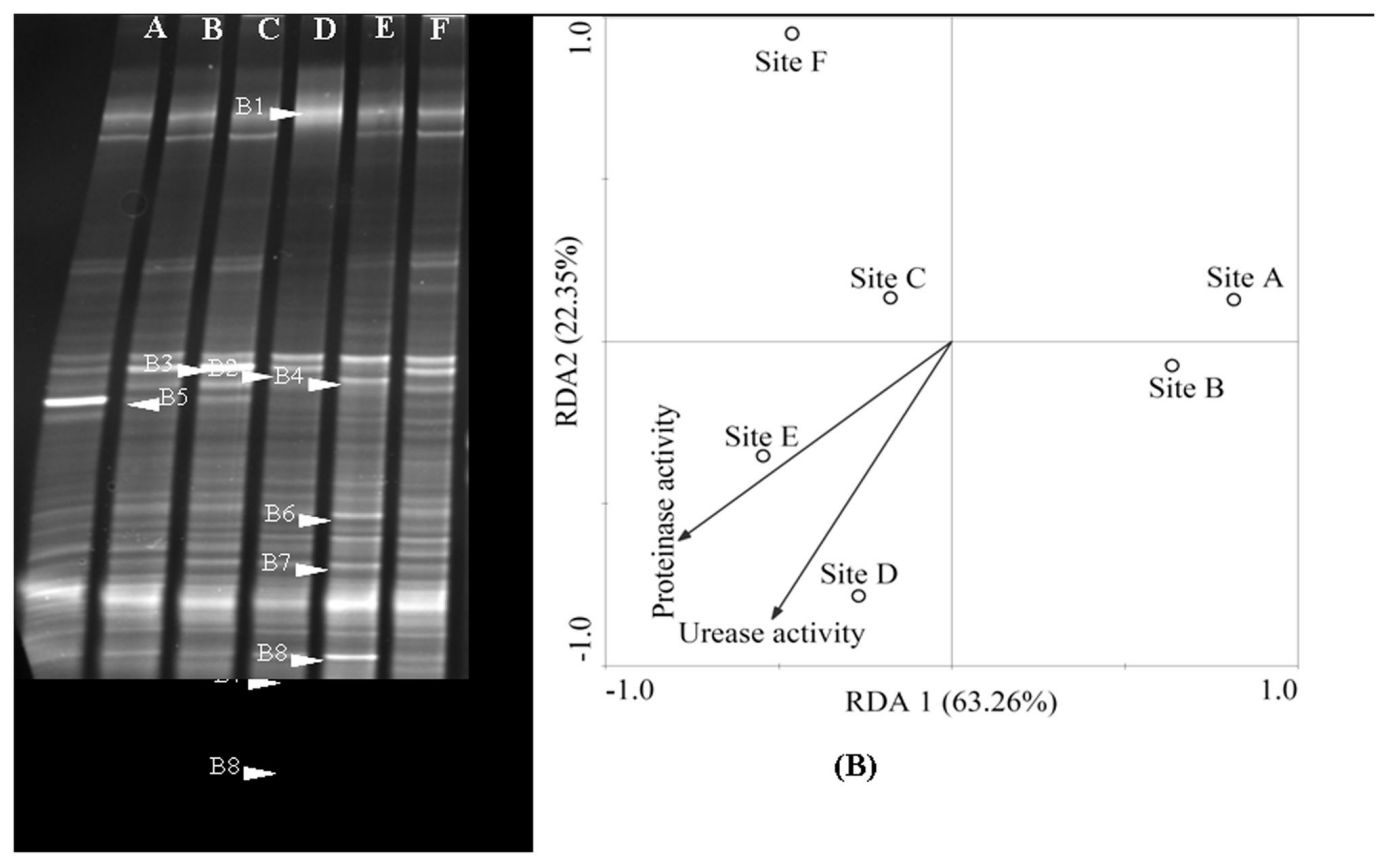
DGGE profiles of PCR-amplified 16S rRNA gene (V3 region). (A) Fragments in a denaturing from 30% to 70% gradient gel. B1-B8 represents sequenced bands in the bacterial community DGGE gel. (B) Redundancy Analysis (RDA) of bacterial community in the sediments from each six sampling sites (site A, site B, site C, site D, site E, site F) in the Zhou cun drinking water reservoir, eastern China. Samples are represented by open circles and the capital letters refer to the sampling sites. Numbers in brackets represent the percentage of variation explained by each factor, RDA1 explains 63.26% of the variance of the data and RDA2 explains 22.35% of the variance in the data, respectively. Significant factors for variation extracted from environmental data (sediment urease and protease activity) are given as vectors.

To understand more specifically of the sediment bacterial and fungal species living in Zhou cun drinking water reservoir, a deeper examination of dominant DGGE band sequences is needed. Figure 4 showed the PCR-DGGE profiles of sediment bacterial community from different sampling sites (Figure 4A). 

**Figure 5:**
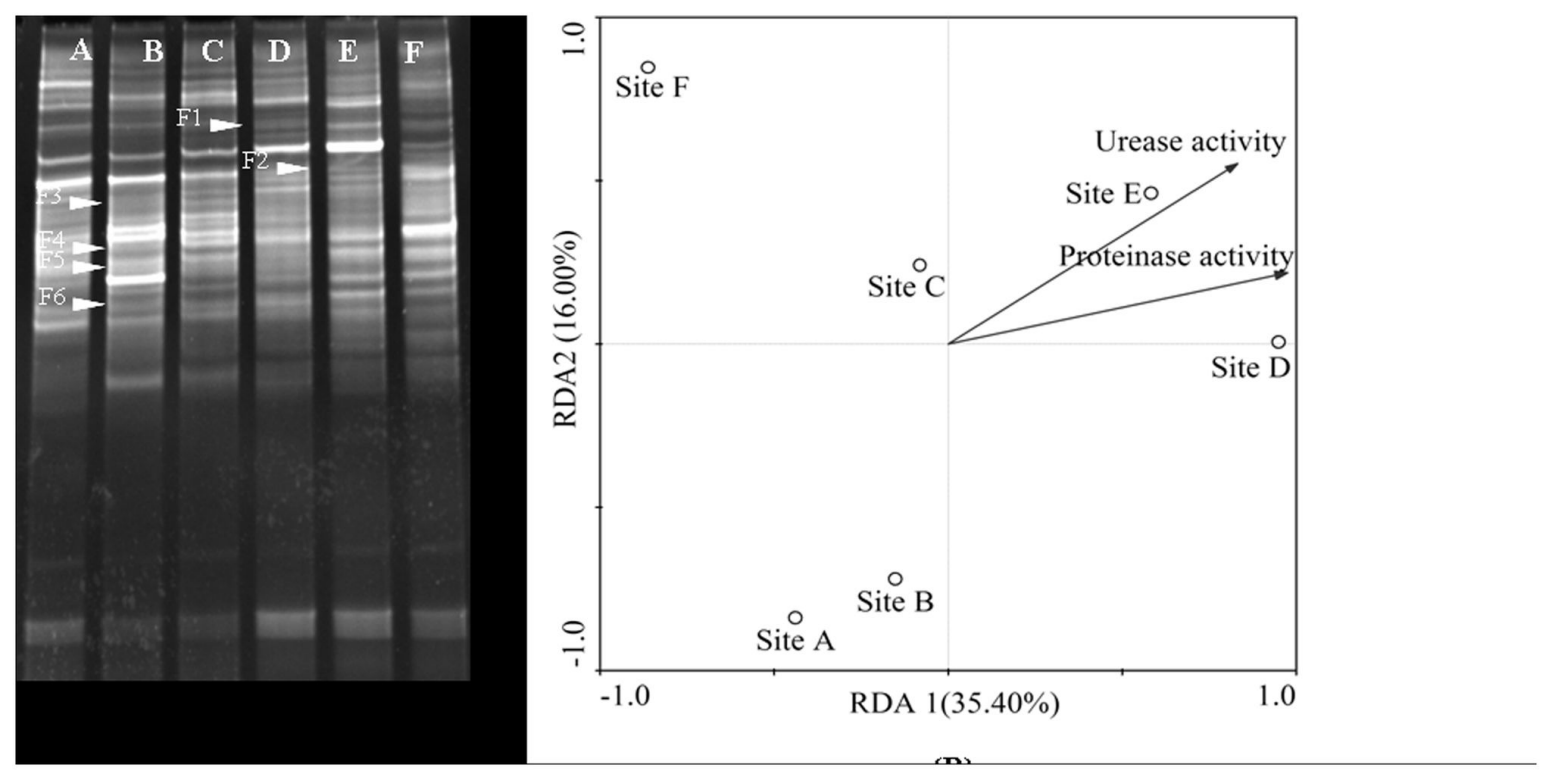
DGGE profiles of PCR-amplified 18S rRNA gene-internal transcribed spacer. (A) Fragments in a denaturing from 30% to 70% gradient gel. F1-F6 represents sequenced bands in the fungal community DGGE gel. (B)Redundancy Analysis (RDA) of fungal community in the sediments from each six sampling sites (site A, site B, site C, site D, site E, site F) in the Zhou cun drinking water reservoir, eastern China. Samples are represented by open circles and capital letters refer to the sampling sites. Numbers in brackets represent the percentage of variation explained by each factor, RDA1 explains 35.40% of the variance of the data and RDA2 explains 16.00% of the variance in the data, respectively. Significant factors for variation extracted from environmental data (sediment urease and protease activity) are given as vectors.

As shown in Figure 4, the first two axis (RDA1 and RDA2) were sufficient to explain near 85.61% of the total variance (Figure 4B). The first axis (RDA1) separated site A, B and site C, D, E and F. In order to identify the bacterial species, the typical dominant bands were excised and sequenced. As shown in Figure 4A, sequenced bands were B1-B8. Eight excised DGGE bands (marked in Figure 4A) had sequences (Table 5). The classification status (LCA Tax. SILVA) were Ruminococcaceae, *Cyanobacteria*, Chloroflexi, *Comamonas* sp., *Syntrophus* sp., Deltaproteobacteria and *Clostridium* sp., respectively. *Comamonas* sp. was the dominant bacterial species in site A. Uncultured *Chloroflexi* sp. bacteria was observed in site C. As shown in Figure 5, sediment fungal community at site A and B differed significantly from those of the other sites (Figure 5). 

**Table 5:** Sequencing and taxonomic affiliation of the excised bands from the DGGE gels of sediment bacterial and fungal species in the Zhou cun drinking water reservoir, eastern China.

Band name	Sequence size (bp)	Accession no.	Closest related organism in the database (accession no.)	Similarity (%)	Taxonomic affiliation (LCA Tax. SILVA)
B1	172	KC775759	Uncultured bacterium isolate 16S ribosomal RNA gene, partial sequence (GU224074.1)	100	Ruminococcaceae
B2	174	KC775760	Uncultured bacterium clone S89 16S ribosomal RNA gene, partial sequence (JX220059.1)	100	*Cyanobacteria*
B3	162	KC775761	Uncultured bacterium clone S89 16S ribosomal RNA gene, partial sequence (JX220059.1)	97	*Cyanobacteria*
B4	170	KC775757	Uncultured *Chloroflexi bacterium* clone G279 16S ribosomal RNA gene, partial sequence (HQ162767.1)	99	Chloroflexi
B5	160	KC775762	*Comamonas* sp. AS-8 16S ribosomal RNA gene, partial sequence (KC013936.1)	100	*Comamonas* sp.
B6	160	KC775763	Uncultured bacterium clone OTU-X4-16 16S ribosomal RNA gene, partial sequence (JQ668617.1)	100	*Syntrophus* sp.
B7	161	KC775764	Uncultured bacterium isolate DGGE gel band S16 16S ribosomal RNA gene, partial sequence (HM118837.1)	99	Deltaproteobacteria
B8	157	KC775765	Uncultured bacterium clone HFMBR bac1-2 16S ribosomal RNA gene, partial sequence (JX853187.1)	100	*Clostridium* sp.
F1	265	KC775766	Uncultured fungus clone KV11a-28-D04 18S ribosomal RNA gene (JX844781.1)	98	Pezizales
F2	276	KC775767	Uncultured fungus clone ITS1-67 18S ribosomal RNA gene (JX915388.1)	100	Glomeromycota
F3	240	KC775768	*Alternaria alternata* TC0811057 18S ribosomal RNA gene (HM013816.1)	100	Deuteromycotina
F4	214	KC775758	Uncultured soil fungus clone BD9 18S ribosomal RNA gene (JQ666632.1)	100	*Rhizophydium* sp.
F5	216	KC775769	*Rhizophydium* sp. JEL-223 (DQ485617.1)	100	Rhizidiaceae
F6	266	KC775770	*Allomyces macrogynus* (JN943673.1)	92	Blastocladiales

The RDA indices of each site indicated that the variation of fungal diversity in sites A and B were similar but different from sites C, D, E and F. Six DGGE bands were re-amplified and sequenced (see number and position in Figure 5A). 

**Figure 6:**
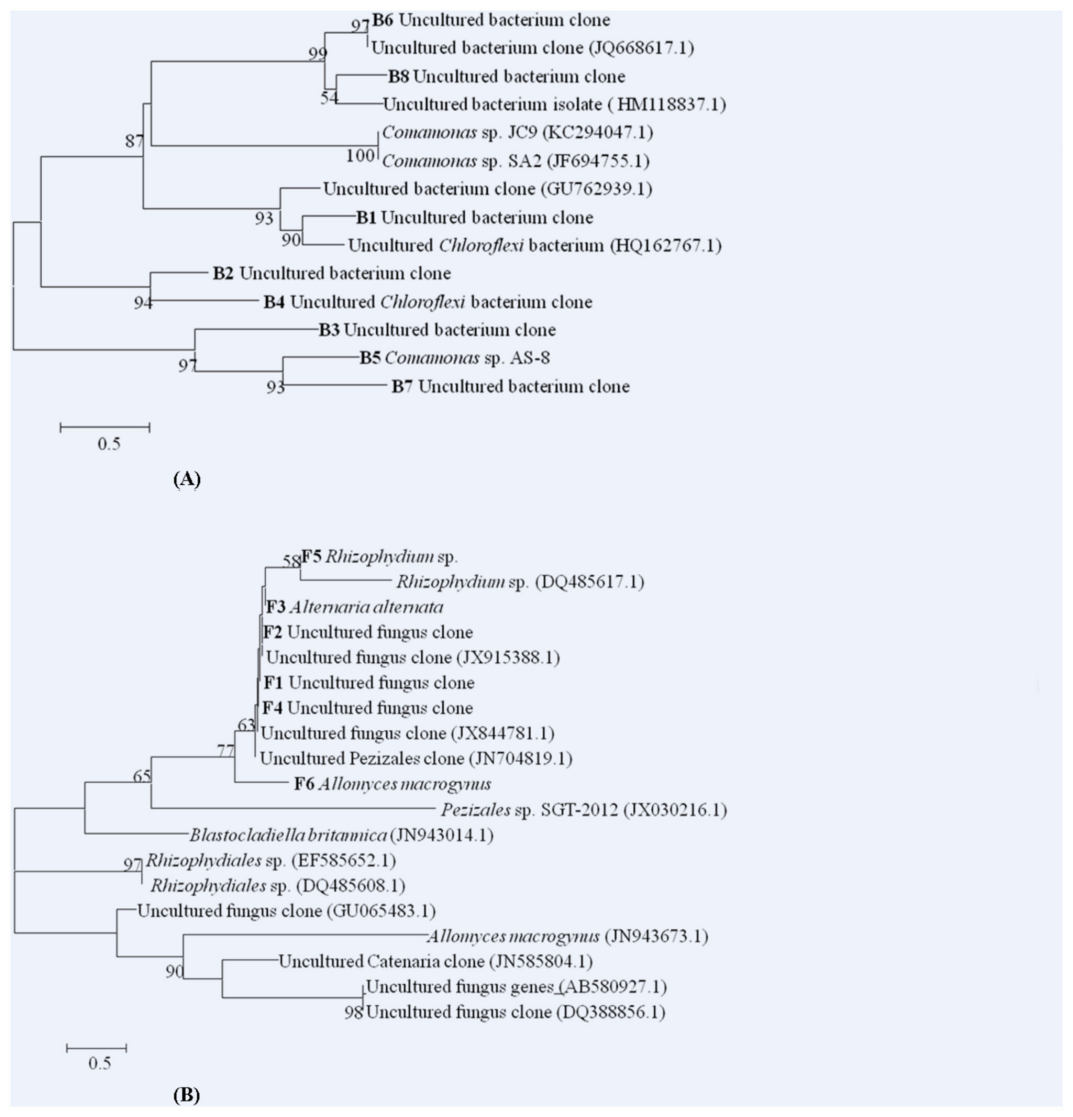
Phylogenetic affiliation of sequences retrieved from sediment (A) bacterial community and (B) fungal community. Bootstrap values (>50%) are indicated at nodes (1000 replications). Sequences obtained in the present study were shown in boldface. The internal letter and number (e.g. B1, F1) represents sequences in the DGGE fingerprints band B1 and F1. Scale bar represents 0.5 substitutions per nucleotide position.

As shown in Table 5, the blasting analysis of the sequences revealed that they were members of the *Alternaria alternate*, *Allomyces macrogynus* and *Rhizophydium* sp., Pezizales, Glomeromycota, and *Rhizophydium* sp., in which *Alternaria alternata* was the most dominant fungal group in sites A, B and C. *Allomyces macrogynus* was dominant fungal species in site B. Uncultured fungus clone was dominant in site E. Redundancy Analysis (RDA) of DGGE profiles showed that urease and protease activities have significantly affected the structural diversity of the sediment bacterial and fungal communities in Zhou cun reservoir (Figure 4, 5). Phylogenetic results showed that B1 and B3 were clustered into the group of uncultured bacteria (Figure 6).

Sediment microbes are important contributors to nutrient cycling and energy transfer in aquatic ecosystems. In the drinking water oligotrophic environmental conditions, the organic matter content input is limited. It is therefore there are lower carbon sources utilized by microbial species. The similar study conducted by Du et al. [31], the catabolic and genetic diversity of microorganisms in the surface sediments at six sites in the Jiaojiang Estuary were determined using BIOLOG and PCR-DGGE methods, suggested that the diversity of sediment microbial catabolism and community structure in Jiaojiang Estuary was affected by estuarine physicochemical conditions. BIOLOG combined with PCR-DGGE techniques were useful for determining aquatic sediment microbial community metabolic and molecular analyses [32]. BIOLOG method was used to explore cultural bacterial community of the soil or sediment; however, many microbial species are belonged to unculturable species, it is therefore molecular methods should be used. In this work, the results showed that urease activity and protease activity were the primary significant environmental factors affecting the structure of sediment microbial communities in Zhou cun drinking water reservoir. Investigation on isolation and characterization of drinking water reservoir sediment oligotrophic bacteria and fungal species is needed in the future. Meanwhile, new techniques should be developed for isolation the oligotrophic microbial species such as oligotrophic aerobic denitrifying bacteria.

## Conclusions

In this study, a culture-dependent method (community level physiological profiles, CLPPs) and a culture-independent technique (PCR-DGGE combined clone sequence) were used to gain a comprehensively understanding on ecological function of sediment enzyme activity and microbial community diversity in Zhou cun drinking water reservoir, eastern China. The results suggested that sediment urease activity was markedly distinct along the sites, ranged from 2.48 to 11.81 mg NH_3_-N/(g·24h). Sediment protease activity was also distinct among the six sampling sites. Utilizations of the carboxylic acids did not significantly change with sampling sites. Principal component analysis (PCA) demonstrated tremendous differences in the functional diversity patterns of the sediment bacterial communities from different sites. Meanwhile, PCR-DGGE fingerprints also revealed spatial changes in genetic diversity of sediment bacterial and fungal communities. *Cyanobacteria*, *Comamonas* sp., *Syntrophus* sp., Deltaproteobacteria and *Clostridium* sp. were found. *Comamonas* sp. was the dominant bacterial species harbored in site A. *Alternaria alternate*, *Allomyces macrogynus* and *Rhizophydium* sp. were most commonly detected fungal species observed in the sediments of the Zhou cun drinking water reservoir, China. Although some progress has been made in this study, the functional microbial species such as anaerobic ammonia oxidation bacteria, sulfate-reducing bacteria and iron-reducing bacteria in the sediment of reservoir should be determined using microarray and high-throughput pyrosequencing in the future. 
